# Multidrug-resistant *Escherichia coli* and *Salmonella* spp. isolated from pigeons

**DOI:** 10.14202/vetworld.2020.2156-2165

**Published:** 2020-10-15

**Authors:** Shah Jungy Ibna Karim, Mahfuzul Islam, Tahmina Sikder, Rubaya Rubaya, Joyanta Halder, Jahangir Alam

**Affiliations:** 1 Department of Medicine and Public Health, Sher-e-Bangla Agricultural University, Dhaka 1207, Bangladesh; 2 Department of Microbiology and Parasitology, Sher-e-Bangla Agricultural University, Dhaka 1207, Bangladesh; 3 Ruminant Nutrition and Anaerobe Laboratory, Department of Animal Science and Technology, Sunchon National University, Suncheon 57922, South Korea; 4 Department of Pathology, Sher-e-Bangla Agricultural University, Dhaka 1207, Bangladesh; 5 Animal Biotechnology Division, National Institute of Biotechnology, Ganakbari, Ashulia, Savar, Dhaka 1349, Bangladesh; 6 Department of Biotechnology and Genetic Engineering, Islamic University, Kushtia 7003, Bangladesh

**Keywords:** antimicrobial resistance, *Escherichia coli*, multidrug resistance, pigeon, *Salmonella* spp

## Abstract

**Background and Aim:**

Pigeon rearing has been gaining popularity for recent years. They are reared remarkably very close to the house of the owner. This activity, therefore, may pose potential threats for humans as well as other animals as pigeons may carry and spread different pathogens including drug-resistant bacteria. This study was conducted to explore the prevalence of *Escherichia coli* and *Salmonella* spp. as well as their antibiogram profile along with an association analysis.

**Materials and Methods:**

Forty swab samples were collected from 20 pigeons during the study. *E. coli* and *Salmonella* spp. were isolated and identified on various types of agars, including MacConkey, Eosin methylene blue, Brilliant green, and Salmonella-Shigella agar. Biochemical tests such as the carbohydrate fermentation test, the triple sugar iron agar slant reaction, the indole test, the methyl red test, the catalase test, as well as the Voges–Proskauer test were also performed. Besides, the presence of *E. coli* was further confirmed by polymerase chain reaction (PCR). Moreover, antimicrobial susceptibility testing of the isolates was performed against nine antibiotics from seven classes on the Mueller-Hinton agar based on the Kirby–Bauer disk diffusion method.

**Results:**

The overall prevalence of *E. coli* and *Salmonella* spp. was 52.5 and 27.5%, respectively. The prevalence of the pathogenic *E. coli* was 61.90%. The antibiogram profile of 21 *E. coli* as well as 11 *Salmonella* spp. revealed that all isolates, except one, were resistant to one to six antibiotics. Around 61.90%, 71.43%, 23.81%, 61.90%, 23.81%, 19.05%, and 52.38% of *E. coli* showed resistance against amoxicillin, ampicillin, azithromycin, erythromycin, nalidixic acid, gentamicin, and tetracycline, respectively. Furthermore, *E. coli* resistance was not observed in case of ciprofloxacin and levofloxacin. Similarly, around 36.36%, 27.27%, 27.27%, 45.45%, 81.82%, 100%, and 18.18% of the *Salmonella* spp. showed resistance against amoxicillin, ampicillin, azithromycin, erythromycin, nalidixic acid, tetracycline, and levofloxacin, respectively. However, all *Salmonella* spp. (100%) were found to show sensitivity against ciprofloxacin and gentamicin. Multidrug-resistant (MDR) *E. coli* (23.80%) and *Salmonella* spp. (54.54%) were also isolated. Furthermore, both positive (odds ratio [OR] >1) and negative (OR <1) drug resistance associations, with a higher frequency of positive associations, were found in *E. coli*. A significant positive association was observed between ampicillin and amoxicillin (OR: 81.67, 95% confidence interval: 2.73-2447.57, p=0.01).

**Conclusion:**

Pigeon carrying MDR *E. coli* and *Salmonella* spp. may contribute to the transmission and spread of these microorganisms. Therefore, strict hygienic measures should be taken during the farming of pigeons to decrease the potential transmission of *E. coli* and *Salmonella* spp. from pigeon to humans as well as other animals. So far, this is the first report of the PCR-based identification of pathogenic *E. coli* from pigeons in Bangladesh.

## Introduction

The Asian subcontinent was a pioneer in rearing fancy pigeons. Emperor Akbar kept 20,000 pigeons where 500 were selected [1] with the primary purpose of using them as postal messengers [2]. Furthermore, a long historical record can be found in Bangladesh in association with raising poultry in the backyard system [3]. Both domestic and feral pigeons (*Columba livia*) are commonly found in the rural as well as urban areas of Bangladesh. The vast spreading of the areas of crop fields and the weather of Bangladesh are suitable for pigeon farming and rearing to provide a source of nutrition for families, recreation, and income generation [4]. In Bangladesh, the most commonly found rock pigeon species is the Indian blue rock pigeon – *C. livia* *intermedia* [5].

Since pigeons are the potential hosts for various microorganisms, including *Salmonella* spp., *Campylobacter*, *Escherichia coli*, and *Chlamydia*, they pose significant threats to humans who remain in close contact with pigeons at their home, live-bird markets, and farms [6-8]. Healthy pigeons are important sources of salmonellosis for humans [9]. The occurrence of common diseases in pigeons in the northern part of Bangladesh varies significantly according to the current season and age of the birds. Younger citizens are more susceptible to salmonellosis and pigeon pox [10]. Moreover, the previous studies reported that bacterial infections (*E. coli*) for multiple species, including pigeons were found in Bangladesh [11]. Furthermore, a higher prevalence of *Salmonella* spp. was noticed in seemingly healthy pigeons and their carcasses that were reared and sold in live-bird markets, farms, and villages [9]. In yards and live-bird markets, the feces of pigeons largely contribute to the spreading of the infectious agents to the surrounding environment. Healthy pigeons may carry *Salmonella* spp., bearing zoonotic importance [12]. Furthermore, the meat of the pigeons might be contaminated with *Salmonella* spp. when it is prepared and kept unhygienically [13]. *E. coli* is one of the most recognized and important foodborne pathogen. Several animal species that are bred to provide food, including chicken, cattle, and pig, appear to be the host of these pathogenic microorganisms [14]. The feces of pigeons are a source of *E. coli* and they are extremely effective transmitters of *E. coli* to humans, birds, and mammals [15]. Regarding humans, a possible zoonotic risk from *E. coli* has been suggested, which triggers the appearance of urinary infections [16-18].

Antimicrobial resistance (AMR) limits the therapeutic possibilities of treatment associated with bacterial diseases in domestic animal species and poultry in particular [19]. Birds may host strains of AMR pathogens and disseminate them, posing a risk to humans [20]. The prevalence of AMR has been on the rise in regard to major bacterial pathogens [21]. Several animal species that are bred to provide food harbor multidrug-resistant (MDR) *Salmonella* spp. and become an emerging issue all over the world. Antibiotic-resistant zoonotic agents in an animal host potentially enter into humans through the food chain [22,23].

This study was, therefore, designed to detect *E. coli* and *Salmonella* spp. from pigeons, especially in association with their antibiogram from seemingly healthy pigeons raised in households and farms in and around Dhaka city.

## Materials and Methods

### Ethical approval and Informed consent

Ethical approval is not required for such type of study. However, pigeons were handled carefully during collection of sample. Prior consent was taken from owner of the pigeon farm. The privacy and confidentiality of personal information of participating farm owners are not disclosed in the manuscript.

### Study period, location, sample collection and preparation

A total of 40 samples, including both oral swabs (n=20) and cloacal swabs (n=20), were collected from 20 pigeons raised in farms and under household conditions around the Savar area as well as Sher-e-Bangla Agricultural University in Dhaka during the period of January-May 2017. One oral and one cloacal swab were collected from each bird subject. Swabs were placed into a tube that contained phosphate-buffered saline (PBS) immediately after the collection, which was transferred to the laboratory for analysis.

### Total viable count

For the calculation of the total viable count, samples were diluted into 10-fold dilution series with sterile PBS. Diluted samples were subsequently cultured in nutrient agar at 37°C. Colonies were counted and the results were expressed as CFU/ml. A portion of the sample was enriched in Luria-Bertani broth at 37°C overnight. Afterward, the total *E. coli* count as well as the total *Salmonella* spp. count (TSC) were plated in MacConkey (MC) agar and Salmonella-Shigella (SS) agar, respectively. Results were expressed as CFU/ml.

### Isolation and identification

The isolation and characterization of *E. coli* and *Salmonella* spp. were performed as previously described [24,25]. *E. coli* and *Salmonella* spp. strains were isolated from the collected samples with a sterilized inoculating loop. The primary culture was plated on nutrient agar. Subcultures were subsequently plated on MC agar, eosin methylene blue (EMB) agar, brilliant green agar, and SS agar to get pure culture and cultural characteristics.

### Morphological characteristics

The isolated *E. coli* and *Salmonella* spp. strains were stained by Gram’s stain according to the procedure described earlier [24].

### Biochemical test

Biochemical tests, including the carbohydrate fermentation test, the triple sugar iron agar slant reaction, the indole test, the methyl red (MR) test, the catalase test, as well as the Voges–Proskauer (VP) test, were performed according to the procedures described previously [24,25].

### DNA extraction

For DNA extraction, individual *E. coli* was cultured in a nutrient broth. About 1.0 mL of the broth cultured overnight was spanned at 12,000 rpm for 3 min. After decanting the supernatant, the bacterial pellet was resuspended in 467 μL TE buffer. Subsequently, 30 μL of 10% sodium dodecyl sulfate (SDS) and 3 μL of proteinase K were added to give a final concentration of 100 μg/ml proteinase K in 0.5% SDS. The mixture was then shaken and incubated for 1 h at 37°C. An equal volume of 500 μL of phenol/chloroform/isoamyl alcohol was added and mixed thoroughly by inverting the tube until the phases got completely mixed. Afterward, the sample was centrifuged at 12,000 rpm for 10 min. Following the centrifugation, the aqueous and viscous supernatant (~450 μL) was transferred to a fresh microcentrifuge tube and an equal volume of phenol/chloroform/isoamyl alcohol was added, mixed, and the mixture was further spanned at 12,000 rpm for 5 min. The supernatant was then transferred to a fresh tube (~400 μL) and about 1/10^th^ volume of 3 M sodium acetate was added. Subsequently, 0.6 volumes of isopropanol were added and kept on ice for 10 min for DNA precipitation. The mixture was then centrifuged at 13,500 rpm for 15 min. The supernatant was decanted, and 1 mL of 95% ethanol was added and kept at room temperature for 5 min. Finally, the mixture was centrifuged at 12,000 rpm for 10 min. After decanting, the supernatant DNA pellet was dried and resuspended in DNase-free water.

### Polymerase chain reaction (PCR)

PCR reactions were performed in a total volume of 25 μL, including 1.5 mM MgCl_2_, 50 mM KCl, 10 mM Tris-HCl (pH 9.0), 0.1% Triton X-100, 200 μM of each dNTP, 1 μM primers (Table-1), 1 unit of Taq DNA polymerase, as well as 100 ng of DNA. A thermocycler was used to carry out amplification reactions (GeneAtlas, Model: G02, Japan) as follows: The initial denaturation was set to 5 min at 95°C, followed by 35 cycles with each cycle consisting of 1 min at 94°C, 90 s at ~55°C, and 1 min at 72°C, and a subsequent final extension was set to 10 min at 72°C [26]. Afterward, electrophoresis was used to analyze amplicons in 1.5% agarose gel stained with ethidium bromide. A molecular weight marker with 100 bp increments (100 bp DNA ladder) was used as a standard. Positive and negative controls were also used along with test samples.

**Table 1:** PCR primers with sequence.

Primer	Sequence (5´-3´)	Size (bp)	Reference
*E. coli* 16E1 (F)	GGGAGTAAAGTTAATC CTTTGCTC	584 bp	[26]
*E. coli* 16E2 (R)	TTCCCGAAGGCACATTCT		
*E. coli* 16E3 (R)	TTCCCGAAGGCACCAATC		

*E. coli=Escherichia coli*, PCR=Polymerase chain reaction

### Antibiotic sensitivity assay

The isolated *E. coli* and *Salmonella* spp. were subjected to an antimicrobial sensitivity test against nine commonly used antibiotics of different groups by the Kirby–Bauer disk diffusion method [27]. Briefly, the overnight grown bacterial inoculums were adjusted to the 0.5 McFarland standard, swabbed on pre-incubated Mueller-Hinton agar (MHA) plates by a sterile cotton swab, and subsequently left for 10-15 min to dry. Afterward, standard antibiotic disks (Oxoid Ltd., U.K.) were placed on MHA plates with sterile forceps and aerobic incubation took place at 37°C for 24 h. Following the incubation, the organisms were categorized as “resistant” or “susceptible” based on the diameter of their zone of inhibition according to the CLSI guidelines [28]. Antibiotic classes and antibiotics chosen for this study include the following compounds: Penicillin ­antibiotics (ampicillin – 10 μg/disk), beta-lactam antibiotics (amoxicillin – 10 μg/disk), macrolides (erythromycin – 15 μg/disk and azithromycin – 15 μg/disk), tetracyclines (tetracycline – 30 μg/disk), quinolones ­(nalidixic acid – 30 μg/disk), fluoroquinolones (ciprofloxacin – 5 μg/disk and levofloxacin – 5 μg/disk), as well as aminoglycosides (gentamicin – 10 μg/disk).

MDR in *Enterobacteriaceae*, including *E. coli*, is defined as being resistant to at least one drug from three or more of the following antimicrobial classes: Aminoglycosides (i.e., gentamicin, tobramycin, amikacin, or netilmicin), fluoroquinolones (i.e., ciprofloxacin), penicillin antibiotics (i.e., ampicillin), tetracyclines (i.e., tetracycline, doxycycline, or minocycline), phenols (i.e., chloramphenicol), and folate pathway inhibitors (i.e., trimethoprim-sulfamethoxazole), among others [29]. Resistant and intermediate sensitive isolates were considered as non-susceptible during the calculation of the MDR phenotype [29].

## Results

### Bacterial load

The bacterial load was determined from 40 swab samples (20 oral and 20 cloacal swabs) obtained from 20 pigeons that were taken from two different regions (Dhaka and Savar) and reared under two different conditions, the household as well as the small farm condition. In general, 100% of the samples were found positive for the total viable count and bacterial load that ranged between 7×10^4^ and 3.8×10^9^ CFU/ml. *E. coli* and *Salmonella* spp. were found in 52.5% and 27.5% of the samples, respectively. Furthermore, the coliform count was found to vary between 8×10^4^ and 6.8×10^9^ CFU/ml, whereas *Salmonella* spp. load varied between 1.3×10^5^ and 3.6×10^9^ CFU/ml (Table-2). In addition, the prevalence rate and bacterial load varied among the selected region, rearing system, and the type of the sample. The total viable count (3.4×10^9^ CFU/ml) and the coliform count (6.8×10^9^ CFU/ml) were found to be higher in the oral swab samples of pigeons reared under the household condition in the Savar region. On the other hand, the TSC was found to be higher (2.7×10^9^ CFU/ml) in the cloacal swab samples of pigeons from the same region (Table-2).

**Table 2:** Total viable count, *Escherichia coli* count, and *Salmonella* spp. count in the swab samples of pigeon (n=40).

Category	Sample type	Number of sample tested	Microorganism count (CFU/ml)					

			Total viable		Total *Escherichia coli*		Total *Salmonella* spp.	
		
			Number of positive (%)	Bacterial load	Number of positive (%)	Bacterial load	Number of positive (%)	Bacterial load
Sample from around Savar								
Household	Oral swab	10	10 (100)	1.6×10^5^-3.8×10^9^	6 (60)	0.8×10^5^-6.8×10^9^	2 (20)	1.6×10^5^-1.8×10^5^
	Cloacal swab	10	10 (100)	1.8×10^5^-3.4×10^9^	5 (50)	3.1×10^6^-5.7×10^9^	2 (20)	1.8×10^5^-2.7×10^9^
Sample from around Sher-e-Bangla Agricultural University, Dhaka								
Small farm	Oral swab	10	10 (100)	0.7×10^5^-3.6×10^9^	4 (40)	1.7×10^9^-6.5×10^9^	4 (40)	1.3×10^5^-3.6×10^9^
	Cloacal swab	10	10 (100)	1.3×10^5^-3.6×10^9^	6 (60)	2.9×10^7^-4.1×10^9^	3 (30)	1.3×10^5^-2.5×10^9^
Overall prevalence		40	40 (100)		21 (52.5)		11 (27.5)	

E. coli=Escherichia coli

### Identification of *E. coli*

*E. coli* sample on the EMB agar produced a greenish-black colony with a metallic sheen, whereas on the MC agar, it produced bright, pink-colored, transparent smooth and raised colonies. Pink-colored, rod-shaped, short chain, single or paired Gram-negative bacilli were observed after the application of the Gram’s staining. The five basic sugars were fermented, including dextrose, sucrose, lactose, maltose, and mannitol, with the production of both acid and gas. Acid production was indicated by a color change from reddish to yellow and gas production was noted by the presence of gas bubbles in the inverted Durham’s tubes. Each *E. coli* isolate was catalase, indole, M-R positive, and V-P negative. Afterward, a molecular identification was conducted.

### Identification of *Salmonella* spp.

*Salmonella* spp. formed smooth, small, round, and black-centered colonies on the S-S agar as well as showed a colorless, smooth, and transparent appearance on the MC’s agar. *Salmonella* spp. showed a structure made of Gram-negative (pink) rods that were arranged in a single form or pairs according to the Gram’s method. The carbohydrate fermentation test showed that *Salmonella* spp. fermented maltose, dextrose, and mannitol and produced both acid and gas, whereas it did not ferment sucrose and lactose. The MR test was positive, whereas the VP and the indole tests were negative.

### Prevalence of *E. coli*

The overall prevalence of *E. coli* was found to be 52.50% (n=21/40). The prevalence was 50% (n=10/20) in oral swabs and 55% (n=11/20) in cloacal swabs (Table-3). Furthermore, the prevalence was recorded to be 55% (11/20) in pigeons reared at farms and 50% (10/20) in pigeons reared at under the household conditions (Table-4).

**Table 3:** Prevalence of *E. coli* and *Salmonella* spp. in oral and cloacal swabs of pigeons.

Sources	Number of sample tested	Number of sample positive	Prevalence (%)
*E. coli*			
Oral swabs	20	10	50
Cloacal swabs	20	11	55
Overall	40	21	52.5
*Salmonella* spp.			
Oral swabs	20	6	30
Cloacal swabs	20	5	25
Overall	40	11	27.5

E. coli=Escherichia coli

**Table 4:** Prevalence of *E. coli* and *Salmonella* spp. in pigeons according to rearing system.

Rearing system	Number of sample tested	Number of sample positive	Prevalence (%)
*E. coli*			
Household	20	10	50
Small farm	20	11	55
Overall	40	21	52.5
*Salmonella*			
Household	20	7	35
Small farm	20	4	20
Overall	40	11	27.5

E. coli=Escherichia coli

### Prevalence of *Salmonella* spp.

The overall prevalence of *Salmonella* spp. was found to be 27.50% (n=11/40). The prevalence was 30% (n=6/20) in oral swabs and 25% (n=5/20) in cloacal swabs (Table-3). Moreover, the prevalence was recorded to be 20% (4/20) in pigeons reared at farms and 35% (7/20) in pigeons reared under the household conditions (Table-4).

### Molecular identification of *E. coli*

**Figure-1:**
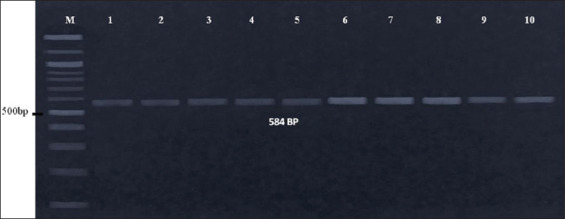
Molecular detection and differentiation of *Escherichia coli*. About 584 bp fragment of 16s rRNA gene was amplified. Lane M: DNA marker, lane 1-5: Test sample, polymerase chain reaction (PCR) with 16E1+E2 primer, lane 6-10: Test sample, PCR with 16E1+16E3 primer.

DNA isolated from 21 *E. coli* samples was subjected to PCR amplification. The 584 bp fragments of the 16S rRNA gene could be amplified from all the isolates, as shown in the representative figure (Figure-1). Furthermore, it was found that 584 bp PCR products could be generated by the primer 16E1+16E2 only from six strains (strain 3, 4, 6, 8, 9, and 10) isolated from pigeons reared under the household conditions and from seven strains (strain 2, 3, 5, 6, 7, 9, and 10) isolated from pigeons reared under the small farm conditions. In addition, these isolates were found to be pathogenic (61.90%). On the other hand, 584 bp PCR products could be generated by the primer 16E1+16E3 only from four strains (strain 1, 2, 5, and 7) isolated from pigeons reared under the household conditions, and four strains (strain 1, 4, 8, and 11) isolated from pigeons reared under the small farm conditions. Furthermore, these isolates were found to be non-pathogenic (38.10%). However, all *E. coli* strains, both pathogenic and non-pathogenic (100%), that were tested generated the expected PCR products of 584 bp when all the three primers (16E1+16E2+16E3) were used together (Table-5).

**Table 5:** Detection of *E. coli* by PCR.

Source of *E. coli*	Number of isolate tested	Detection of different *E. coli* by primer set		

		16E1+16E2 pathogenic	16E1+16E3 non-pathogenic	16E1+16E2+16E3 both pathogenic and non-pathogenic (%)
Household	10	6 (60.00)	4 (40.00)	100 (100)
Small farm	11	7 (63.64)	4 (36.36)	11 (100)
Overall	21	13 (61.90)	8 (38.10)	21 (100)

*E. coli=Escherichia coli*, PCR=Polymerase chain reaction

### Antibiotic sensitivity

Twenty-one *E. coli* isolates from the pigeon samples were subjected to antibiogram profiling with nine antibiotics. Regarding the results, all the tested bacteria (100%) were found to be sensitive to ciprofloxacin and levofloxacin. However, about 61.90%, 71.43%, 23.81%, 61.90%, 23.81%, 19.05%, and 52.38% of *E. coli* isolates were found to be resistant to amoxicillin, ampicillin, azithromycin, erythromycin, nalidixic acid, gentamicin, and tetracycline, respectively. Similarly, *Salmonella* spp. (n=11) isolates from pigeon were subjected to antibiogram profiling and about 36.36%, 27.27%, 27.27%, 45.45%, 81.82%, 100%, and 18.18% of *Salmonella* spp. isolates were found to be resistant to amoxicillin, ampicillin, azithromycin, erythromycin, nalidixic acid, tetracycline, and levofloxacin, respectively. Moreover, all *Salmonella* spp. (100%) isolates were found to be sensitive to ciprofloxacin and gentamicin. These results varied according to the type of the sample and the rearing system (Table-6).

**Table 6:** Antibiogram profile of *E. coli* and *Salmonella* spp. to different antimicrobials.

Name of samples	Number of isolates tested	Number and percentage of microorganisms resistant to antimicrobials								

		AMX	AMP	AZM	ERY	NA	CIP	GM	TET	LEV
*E. coli*										
Oral swabs	10	4 (40)	6 (60)	4 (40)	5 (50)	4 (40)	0 (0)	2 (20)	7 (70)	0 (0)
Cloacal swabs	11	9 (81.81)	9 (81.81)	1 (9.09)	8 (72.72)	1 (9.09)	0 (0)	2 (18.18)	4 (36.36)	0 (0)
Total	21	13 (61.90)	15 (71.43)	5 (23.81)	13 (61.90)	5 (23.81)	0 (0)	4 (19.05)	11 (52.38)	0 (0)
*Salmonella* spp*.*										
Oral swabs	6	2 (33.33)	2 (33.33)	2 (33.33)	3 (50)	5 (83.33)	0 (0)	0 (0)	6 (100)	2 (33.33)
Cloacal swabs	5	2 (40)	1 (20)	1 (20)	2 (40)	4 (80)	0 (0)	0 (0)	5 (100)	0 (0)
Total	11	4 (36.36)	3 (27.27)	3 (27.27)	5 (45.45)	9 (81.82)	0 (0)	0 (0)	11 (100)	2 (18.18)

AMX=Amoxicillin, AMP=Ampicillin, AZM=Azithromycin, ERY=Erythromycin, NA=Nalidixic acid, CIP=Ciprofloxacin, GM=Gentamicin, TET=Tetracycline, LEV=Levofloxacin, (zone diameter interpretive standard [mm] resistant ≤13, intermediate 14-17, susceptible ≥18), *E. coli=Escherichia coli*

### MDR bacteria

In general, about 95.24% (20/21) of *E. coli* isolates and 100% (11/11) of *Salmonella* spp. isolates were found to be resistant to at least one of the antibiotics used in this study. Only one *E. coli* isolate was found to be sensitive to all the tested antibiotics. Microorganisms that showed resistance to three or more antibiotics of three different classes were considered to be MDR. However, macrolides were not included in the MDR calculations. Overall, about 23.80% and 54.54% of *E. coli* and *Salmonella* spp. isolates were found to carry MDR traits, respectively. With the inclusion of the macrolides, a maximum of six antibiotics were found to be ineffective against one isolate of *E. coli*. However, without the application of macrolides, a resistance against 3-4 antibiotic compounds was found in MDR isolates (Table-7).

**Table 7:** Multidrug-resistant *E. coli* (n=21) and *Salmonella* spp. (n=11) isolated from pigeons.^a^

Antimicrobial compound	Antibiotic class	Number of MDR isolates (%)
*E. coli* (n=21)		
AMP, NA, GM	Pen-Qui-Ami	1 (4.76)
AMP, AMX, GM	Pen-Bet-Ami	1 (4.76)
AMP, AMX, NA, TET	Pen-Bet-Qui-Tet	1 (4.76)
AMP, AMX, GM, TET	Pen-Bet-Tet-Ami	1 (4.76)
AMP, NA, GM, TET	Pen-Qui-Tet-Ami	1 (4.76)
Total		5 (23.80)
*Salmonella* spp. (n=11)		
AMP, NA, TET	Pen-Qui-Tet	2 (18.18)
AMX, NA, TET	Bet-Qui-Tet	2 (18.18)
NA, TET, LEV	Qui-Tet-Flu	1 (9.09)
AMP, NA, TET, LEV	Pen-Qui-Tet-Flu	1 (9.09)
Total		6 (54.54)

a MDR was calculated according to Magiorakos *et al*. [29]. Hence, macrolides are not included in MDR calculation. AMX=Amoxicillin, AMP=Ampicillin, AZM=Azithromycin, ERY=Erythromycin, NA=Nalidixic acid, CIP=Ciprofloxacin, GM=Gentamicin, TET=Tetracycline, LEV=Levofloxacin, Ami=Aminoglycosides, Pen=Penicillins, Tet=Tetracyclines, Bet=Beta-lactams, Qui=Quinolones, Flu=Fluoroquinolones, *E. coli=Escherichia coli*, MDR=Multidrug-resistant

### Associations among AMR phenotypes

Phenotypic resistance to each of the applied drugs was found to be probably associated with phenotypic resistance to another drug (Table-8). Both positive (odds ratio [OR] >1) and negative (OR <1) associations were identified in this study, among which higher frequencies of positive associations were observed for *E. coli* than *Salmonella*. In the case of *E. coli*, a significant positive association of AMR was observed between the ampicillin/amoxicillin antibiotic pair (OR: 81.67%, 95% CI: 2.73-2447.57, p=0.01). Besides, a statistically non-significant positive association was also revealed between the amoxicillin/tetracycline (OR: 16, 95% CI: 0.96-267.05, p=0.06), the amoxicillin/gentamicin (OR: 15.93, 95% CI: 0.74-345.09, p=0.08), as well as the azithromycin/tetracycline (OR: 7.64, 95% CI: 0.81-72.41, p=0.08) antibiotic pairs. In the case of *Salmonella*, non-significant positive associations were detected. Remarkable non-significant positive associations include the resistance for tetracycline/nalidixic acid (OR: 23, 95% CI: 0.19-2896.22, p=0.21), ampicillin/ciprofloxacin, (OR: 9.30, 95% CI: 0.35-252.47, p=0.19), and amoxicillin/gentamicin (OR: 8, 95% CI: 0.51-127.91, p=0.15).

**Table 8:** Pairwise association of antibiotic resistance phenotypes in *Escherichia coli* (lower panel) and *Salmonella* spp. (upper panel) isolated from pigeon.^a^

	AMP	AMX	ERY	AZM	TET	NA	CIP	LEV	GEN
AMP	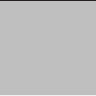	0.34	0.80	1.50	0.85	0.85	9.30	3.00	0.13
		(0.03-3.93)	0.04-17.20)	(0.14-16.55)	(0.02-50.11)	(0.02-50.11)	(0.35-252.47)	(0.26-35.34)	(0.01-1.10)
		0.40	0.89	0.75	0.94	0.94	0.19	0.39	0.15
AMX	81.67	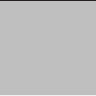	0.17	3	1.19	1.19	0.11	0.34	8
	(2.73-2447.57)		(0.01-4.36)	(0.26-35.34)	(0.020-69.99)	(0.020-69.99)	(0.01-2.93)	(0.03-3.93)	(0.51-127.91)
	0.01		0.28	0.39	0.94	0.94	0.19	0.39	0.15
ERY	5.28	3.89	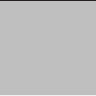	1.25	3.80	3.80	1.67	0.11	4.10
	(0.09-313.60)	(0.07-224.24)		(0.06-26.88)	(0.06-243.53)	(0.06-243.53)	(0.06-47.84)	(0.01-2.93)	(0.16-108.95)
	0.43	0.52		0.89	0.53	0.53	0.77	0.19	0.40
AZM	0.25	0.14	0.63	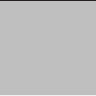	1.19	1.19	0.34	0.34	1.50
	(0.02-3.35)	(0.02-1.68)	(0.02-34.83)		(0.02-69.98)	(0.02-69.98)	(0.03-3.93)	(0.03-3.93)	(0.14-16.55)
	0.30	0.13	0.83		0.94	0.94	0.39	0.39	0.75
TET	4	16	5.29	1.28	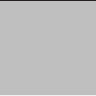	23	0.27	0.85	0.85
	(0.24-66.77)	(0.96-267.05)	(0.09-313.60)	(0.09-16.81)		(0.19-2896.22)	(0.01-16.87)	(0.02-50.11)	(0.02-50.11)
	0.34	0.06	0.43	0.86		0.21	0.53	0.94	0.94
NA	0.47	0.34	3.89	2.10	0.47	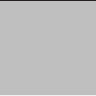	0.27	0.85	0.85
	(0.02-10.71)	(0.02-7.48)	(0.07-224.24)	(0.18-24.60)	(0.02-10.71)		(0.01-16.87)	(0.02-50.11)	(0.02-50.11)
	0.63	0.49	0.52	0.56	0.63		0.53	0.94	0.94
CIP	1.28	0.55	0.63	5.56	1.28	2.10	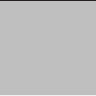	1.25	0.17
	(0.10-16.81)	(0.07-4.92)	(0.02-34.83)	(0.81-38.17)	(0.10-16.81)	(0.18-24.60)		(0.06-26.88)	(0.01-4.36)
	0.86	0.59	0.83	0.08	0.86	0.56		0.89	0.28
LEV	0.20	0.11	0.52	3.34	0.05	6.43	1.35	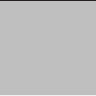	0.13
	(0.02-2.63)	(0.01-1.29)	(0.01-28.76)	(0.51-22.15)	(0.01-1.04)	(0.30-138.26)	(0.22-8.62)		(0.01-1.10)
	0.22	0.08	0.75	0.22	0.06	0.24	0.76		0.15
GEN	10.74	15.93	1.10	1.95	2.5	0.08	0.86	1.35	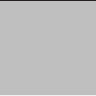
	(0.49-238.92)	(0.74-345.09)	(0.02-60.30)	(0.33-11.76)	(0.20-32.81)	(0.01-1.71)	(0.15-5.0)	(0.22-8.62)	
	0.14	0.08	0.97	0.47	0.49	0.11	0.87	0.76	

a Values in each cell: Before the parenthesis is odds ratio, within the parenthesis is 95% CI and after parenthesis is p value. AMP=Ampicillin, AMX=Amoxicillin, ERY=Erythromycin, AZM=Azithromycin, TET=Tetracycline, NA=Nalidixic acid, CIP=Ciprofloxacin, LEV=Levofloxacin, GM=Gentamicin, CI=Confidence interval

## Discussion

Domestic and feral pigeons (*C. livia*) were commonly found in rural and urban areas of Bangladesh throughout the history of the region. The tradition of pigeon rearing in the Indian subcontinent can be traced back to the Mughal era when pigeons were primarily used as postal messengers. Nowadays, pigeons are commonly raised for racing, fighting, exhibition, as well as for the nutritional and therapeutic values of the pigeon meat [2]. Pigeon farming is gaining popularity among students because only a low level of investment is needed, less technical complexity is required, the requirements in association with space for rearing are low, the housing of pigeons is simple, their marketing is easy, as well as the maximum profit can be high, and so on. However, it must be ensured that pigeons do not get afflicted with diseases, especially as pigeons are mostly reared in and around the same house as their owner. Besides, humans can get into contact with pigeons with a high possibility in smallholdings, parks, temples, shrines, public gardens, and railroad stations [30]. Therefore, they may transmit disease agents to other birds as well as their handlers. Moreover, the live-bird market, the trading place of different species of live poultry, including pigeons, may play a significant role in the transmission of the associated microorganisms. Similar to the diseases associated with chickens, pigeons of Bangladesh are affected with salmonellosis and colibacillosis. The present research aimed to isolate, identify, and characterize *E. coli* and *Salmonella* spp. from pigeons raised in under the household and small farms conditions around the Savar region and the Sher-e-Bangla Agricultural University, Dhaka, with the molecular identification of *E. coli* based on PCR. Forty samples were analyzed in this study and the prevalence was found to be 52.5% (21/40) for *E. coli* isolates (Tables-3 and 4). The prevalence is slightly lower than the prevalence reported by Dey *et al*. [31]. In that study, 112 samples obtained from seemingly healthy pigeons of different places of the Mymensingh district were analyzed and 69.64% prevalence of *E. coli* was reported. Besides, a cross-sectional study on 108 dead pigeons was conducted to explore the diseases and conditions associated with the death of pigeons reared in different regions at smallholdings which were sold at live-bird markets in Bangladesh. In this study, results showed that 13.89% of all deaths were accounted to colibacillosis [30]. Sample size and regional variation, among other factors, may be important factors that contributed to the differences among the results observed in various studies. Moreover, recorded colony characteristics of *E. coli* isolates placed onto EMB agar, MC agar, and SS agar, as well as staining, and biochemical properties were in agreement with the findings of other authors reported elsewhere [32-34].

In our study, the prevalence of *Salmonella* spp. was found to be 27.5% (Tables-3 and 4). A study on pigeon diseases at Khulna Sadar and the surrounding private farms was previously conducted [10] and reported 20.32% salmonellosis, in general, where more cases were detected among younger (30-90 days of age) pigeons. Hosain *et al*. [9] examined salmonellosis in pigeons from the Mymensingh district and reported 22.22%, 58.33%, and 27.50% prevalence obtained from the cloacal swabs, footpads, and the feces of the pigeons, respectively. However, the overall prevalence of *Salmonella* spp. was 35.71%. This study also reported a variable prevalence of 40.48%, 20.00%, and 30.00% in markets, farms, and villages, respectively. The prevalence of *Salmonella* spp. in samples taken from seemingly healthy quails from Mymensingh was reported to be 13.33% [35]. Furthermore, a study on 400 pigeon samples was conducted in Egypt and reported 5%, 3.5%, and 4.6% prevalence of *Salmonella* spp. in squabs, pigeon, and environmental samples, respectively [36].

Although most strains of *E. coli* are harmless and have a common habitat in the digestive tract, some strains can cause diseases, including foodborne illness, in humans. The findings of the present study showed that most of *E. coli* strains are pathogenic (Table-5). The distribution of pathogenic and non-pathogenic strains is almost similar among the pigeons reared in households and small farms. Tsen *et al*. [26] reported the primers for the detection of pathogenic and non-pathogenic *E. coli*. Primer 16E1 is regarded as the forward and the 16E2/16E3 as the reverse primer. PCR products obtained by 16E1+16E2, 16E1+16E3, and 16E1+16E2+16E3 are of the same 584 bp size. *E. coli* could not be serotyped in the current study, however, in a separate study conducted by Dutta *et al*. [37], 150 samples obtained from pigeons were investigated and 91 *E. coli* (prevalence 60.67%) strains were isolates, where the majority of *E. coli* strains was reported to be pathogenic, including the O157 (9.98%) strain, followed by the O68, O121 (7.69%), O9, O75, O131 (5.49%), O2, O13, and O22 (3.30%) strains. Strains that belonged to serogroup O157 were reported to be verocytotoxigenic [38]. Hence, it is assumed that *E. coli* strains circulating in pigeons may cause diarrhea and other illnesses when a potential exposure is created. It is exceedingly difficult to explain the high prevalence of tested bacteria in oral swab samples of healthy pigeons. However, it should be mentioned that pigeons were generally reared in boxes or cages in highly dense areas. The feeder and drinker were placed in the same box. Moreover, the pigeons may have also picked spilled feed from the bedding materials. Therefore, there is a possibility that the droppings of the pigeons were mixed with the feed water and the bedding materials which resulted in the high prevalence of *E. coli* in the oral swabs as well as in the oral–fecal transmission of bacteria [39-41].

Treatment with one antibiotic may be in association with the development of resistance against another antibiotic due to cross-resistance and co-selection [42,43]. Moreover, a given antibiotic may not only contribute to selective resistance against that same antibiotic, the process of which is called “selection,” but also to the development of resistance against other antibiotics, a process termed as “co-selection.” It is reported that amoxicillin is associated with increased resistance against amoxicillin as well as ciprofloxacin in *E*. *coli* [44]. Besides, the frequent use of trimethoprim causes higher levels of resistance to both ciprofloxacin and nitrofurantoin in *E*. *coli*. The use of amoxicillin and trimethoprim was also reported to be associated with the development of resistance against ciprofloxacin, possibly due to co-selection [44]. Therefore, it can be concluded that the relationship between antibiotic use and antibiotic resistance is complex [44]. In our study, both positive and negative associations of AMR were identified, although higher frequencies of positive associations were observed in the case of *E. coli* isolates compared to *Salmonella* spp. isolates. Furthermore, a strong positive association was found between ampicillin and amoxicillin resistance that may be due to cross-resistance between these two drugs, as reported earlier [42]. Moreover, collateral sensitivity was reported previously in regard of resistance against azithromycin and sensitivity to nalidixic acid among a pathogenic *E*. *coli* strain [45]. However, collateral sensitivity could not be observed between these two antibiotics in the current study, which makes it difficult to explain the reason. AMR is a global problem and poses serious public health concerns. One of the major causes of AMR is the indiscriminate use of antibiotics. Bacteria can resist the action of drugs used for different treatments as they produce various enzymes and metabolites that either degrade the antimicrobial agents or help the bacteria survive through various mechanisms. In the present study, nine different antibiotics were used to perform the antibiogram profiling of *E. coli* and *Salmonella* spp. isolated from pigeons using the disk diffusion method. Almost all *E. coli* as well as all *Salmonella* spp. were found to be resistant to at least one antimicrobial compound used in this study (Table-6), along with a considerable portion of *E. coli* isolates as well as all *Salmonella* spp. isolates showing MDR traits (Table-7). The fact that MDR strains were found in these birds could be explained by their eating habits. Pigeons can acquire pathogens through food and/or water contaminated with human feces and farm waste. This indicates the possible transmission of *E. coli* between birds and humans [46]. Our findings are almost similar to the findings reported earlier [9,47]. Except for levofloxacin, all the antibiotics are randomly used in pigeons in Bangladesh. The emergence of MDR *E. coli* with the involvement of co-resistance to three or more different antibiotic classes was reported and reviewed previously [48,49]. Our findings also comply with those prior findings. Keeping this as well as the human-animal-environment interface in mind, our findings suggest that pigeons may contribute to the transfer and spread of microorganisms, as well as antibiotic-resistant bacteria.

## Conclusion

*E. coli* and *Salmonella* spp. were isolated and identified from seemingly healthy pigeons, including pathogenic *E. coli* isolates, with PCR amplification. The overall prevalence of *E. coli* and *Salmonella* spp. was 52.5% and 27.5%, respectively. The antibiogram study revealed a varying degree of resistance to commonly used antibiotics. Besides, it was found that MDR *E. coli* and *Salmonella* spp. circulated in seemingly healthy pigeons raised in households and small farms around Dhaka city. Therefore, seemingly healthy pigeons that are reared in households and small farms can host MDR *E. coli* and *Salmonella* spp. that may be transmitted to humans as well as other livestock and poultry species.

## Authors’ Contributions

SJIK conceptualized the study, did the sample collection, carried out laboratory analysis, data analysis, manuscript preparation, and revision. MI supervised and revised the manuscript. TS, RR, and JH did the laboratory analysis. JA conceptualized and designed the study, provided diagnostic reagents, supervised, revised the manuscript, and submitted the manuscript. All authors have read and approved the final manuscript.
